# Assessment of arterial whole blood redox potential during cardiopulmonary bypass

**DOI:** 10.1371/journal.pone.0324437

**Published:** 2025-05-27

**Authors:** Vincent Pey, Marion Stephan, Pierre Gros, Cédric Dray, Fanny Bounes, Bertrand Marcheix, Vincent Minville, Anne Galinier, François Labaste

**Affiliations:** 1 RESTORE Research Center, University Toulouse 3-Paul Sabatier, INSERM, CNRS, EFS, ENVT, Toulouse, France; 2 Department of Anaesthesiology and Critical Care, University Hospital of Toulouse, University Toulouse 3-Paul Sabatier,; 3 Laboratoire de Génie Chimique, Université de Toulouse, CNRS, INPT, UPS, Toulouse, France; 4 Department of Cardiovascular Surgery, University Hospital Rangueil, Toulouse, France; 5 Department of biochemistry, Toulouse University Hospital, Toulouse, France; South Valley University Faculty of Medicine, EGYPT

## Abstract

**Introduction:**

Imbalance in the redox equilibrium is common in any type of aggression. Cardiopulmonary bypass (CPB) initiation induces metabolic perturbations, and reliable biological monitoring tools for this condition are currently limited (e.g., lactate/pyruvate ratio). The measurement of arterial whole blood redox potential (E_redox_) provides a systemic assessment of the redox state and may serve as a valuable marker for detecting metabolic perturbations during CPB. In this prospective exploratory study involving patients undergoing cardiac surgery, we investigated variations in E_redox_ and lactate/pyruvate ratio during CPB initiation.

**Methods:**

Using a prospective exploratory study design, we assessed the changes in E_redox_ and relevant variables during the initiation of CPB in 16 cardiac surgery patients.

**Results:**

Upon initiation of CPB we observed a significant decrease in arterial whole blood redox potential (101.90 mV + /- 11.52 vs. 41.80 mV + /- 10,26; p < 0.0001). Concomitantly, the lactate/pyruvate ratio significantly increased (12.81 + /- 0.90 vs 67.1 + /- 7.94; p < 0.0001) while the acetoacetate/β-hydroxybutyrate ratio significantly decreased (1.11 + /- 0.19 vs. 0.54 + /- 0.05 at 0 min; p = 0.0055). The circulatory failure indicated by changes in the lactate/pyruvate ratio and ketone bodies at the initiation of CPB correlated with a significant reduction in E_redox_.

**Conclusion:**

Arterial E_redox_ is a novel variable that holds promise in the detection and monitoring of metabolic aggression during CPB. Its assessment during CPB initiation could provide valuable insights into the patient’s circulatory status, as the E_redox_ appears to be more sensitive than lactate for monitoring circulatory insufficiency.

## Introduction

In the operating room, patients undergo multiple insults related to surgery, anesthesia, and the adverse effects of therapeutics. Many of these insults ultimately disrupt cellular metabolism and, among other things and obligatory, the balance between oxidant and reductant species. Measuring specific markers of these imbalances, such as blood lactate levels is well established and is widely used, which refers to tissue hypoxia. However, the causes of these biologic changes can be manifold. Thus, there are non-hypoxic causes for lactate elevation, such as hepatic dysfunction, pyruvate dehydrogenase dysfunction, and accelerated glycolysis [1,2]. Moreover, lactate clearance may also be impaired even after the resolution of the underlying process. As a result, monitoring tissue hypoxia through lactate measurement may lead to misinterpretation. Thus, global redox potential (E_redox_) represents a novel and relevant option for monitoring tissue hypoxia.

The E_redox_ allows to evaluate the balance between oxidized and reduced species by measuring the electron charge of a complex biological environment. In an in vivo model of hemorrhagic shock in pigs, arterial E_redox_ measurement has been correlated with the severity of circulatory failure, elevation of lactate levels, and perturbation of the redox balance in the tissues [3,4]. The E_redox_, unlike lactate, appears to be more sensitive, with a measurement dynamic correlated to the degree of circulatory failure and its management.

There are no human models in the literature that allow evaluation of E_redox_ during circulatory failure. To investigate E_redox_ variation following the circulatory failure in human, we choose the model of the initiation of CPB. Indeed, macro-circulatory and micro-circulatory disturbances during CPB initiation alter the efficiency of mitochondrial oxidative phosphorylation and promote the accumulation of reduced metabolites, such as NADH and lactate [5,6]. These metabolites impair cellular function and activate cell death programs, ultimately leading to organ dysfunction [7]. This model of programmed circulatory failure appears relevant as it allows us to measure the E_redox_ value prior to the onset of circulatory failure. Thus, our objective is to describe the evolution of arterial redox potential during CPB.

## Methods

### Study description

This was a prospective, observational, monocentric study conducted at the Cardiac Surgery Department of the University Hospital Center (CHU) of Toulouse between January and May 2022. The study was approved by an ethics committee (NCT04907565; RC31/21/0014). Informed consent was obtained from the patients before their inclusion, as required by ethical committee and French legislation.

### Population

Patients included were over 60 years old, able to provide informed consent, and scheduled for cardiac surgery under general anesthesia requiring CPB. They were excluded if they required emergency surgery, hypothermia, circulatory arrest or surgeries linked to a septic condition.

### Research procedure

Preoperative preparation followed national recommendations and remained unchanged [8]. Upon arrival in the operating room, each patient underwent standard monitoring, as well as the placement of a central jugular venous line and a radial arterial catheter. After general anesthesia, the patient was mechanically ventilated. All surgeries were performed via sternotomy. Following patient heparinization, CPB was initiated. The blood circulating in the CPB system was warmed (37°C), oxygenated (target pO_2_ at 140 mmHg), and gradually returned to the aorta with increasing flow through the arterial cannula. Once CPB reached the target flow rate (2.4 ml/min/m^2^), cardiac activity was gradually stopped (using cardioplegia consisting in hyperkalemic fresh blood). Immediate postoperative care was provided in the intensive care unit and the cardiac surgery ward, as soon as the patient’s condition allowed. At the end of hospitalization, clinical data were collected.

During the procedure, 2 ml of blood were drawn from the radial arterial catheter. Blood pH, arterial oxygen saturation (SaO_2_), and hemoglobin concentration were immediately measured using a Siemens RP 500 blood gas analyzer. Then, the remainder of 1 ml of blood was mixed with 1 ml of 1N perchloric acid for delayed measurement of redox point (lactate, pyruvate, betahydroxybutyrate, acetoacetate) using BIOSENTEC kits on a ThermoScientific INDIKO+ analyzer.

E_redox_ measurement was performed immediately after blood sampling with a potentiostat (µAutolab type II, Metrohm) using a single-use “lab-made” working platinum microelectrode [9] and a commercialy available reference electrode (BioLogic, Seyssinet-Pariset, France). Both electrodes were immersed in the patient’s arterial whole blood without pre-treatment. Data were collected for 60 seconds using the NOVA® 2.0 application with one potential value recorded per millisecond. The median, the final E_redox_ value, is calculated from the remaining adequate potential values, expressed in millivolts.

During CPB, central venous oxygen saturation (ScVO_2_) was measured by oximetry on the CPB circuit. Based on these measured variables and CPB blood flow (Qc), the oxygen delivery to the tissues (DO_2_ = Qc x 1.34 x [Hb] x SaO_2_), oxygen consumption (VO_2_ = (SaO_2_ - ScVO_2_) x Qc x 1.34 x [Hb]), and oxygen extraction from the tissues (EO_2_ = VO_2_/DO_2_) were calculated.

Samples were collected after induction of general anesthesia before surgical incision (Pre-CPB), at the achievement of the target CPB flow (0 min), at 10, 20 and 30 minutes.

#### Statistical analysis.

Binary variables were expressed as percentages. Continuous variables were expressed as mean and standard error. Statistical analyses were performed using GraphPAD Prism® version 8 software. Univariate statistical tests were conducted using the Mann-Whitney method for continuous variables and the Fisher’s test for binary variables. A p-value < 0.05 was considered statistically significant.

## Results

### Study population

During the study period, 21 patients were included in the study. Sixteen patients had an E_redox_ measurement during CPB. For 5 of them, a technical difficulty prevented obtaining a reliable E_redox_. Lactate and pyruvate measurements were performed for 14 patients with minimal missing data (3.6% - broken tubes during centrifugation, uncertain dilution factor induced by pre-analytical step).

Patient characteristics are summarized in Table 1. The male-to-female ratio among the included patients was 11:5. They had a median age of approximately 71.5 years (Q1-Q3: 67.5–74 y.o.). Most surgeries were coronary artery surgeries (n = 6). Four patients underwent valve surgery, and 2 had aortic surgery. Three procedures involved 2 interventions (valve replacement and bypass). The mean duration of CPB was 70.5 minutes (Q1-Q3: 54.7–80.7 min). All patients in the cohort received norepinephrine during the operation, but none of them required the addition of a postoperative inotrope or circulatory support. Two patients received transfusions during their hospitalization, none during surgery. In the postoperative period, the SAPS II score was approximately 29 (Q1-Q3: 27–37).

**Table 1:** Cohort characteristics.

	Total patients n = 16
a) **Preoperative Data**	
Age (y.o), médiane (Q1-Q3))	71.5 (67.5-74)
Sex:	
Female, No. (%)	5 (31.2)
Male, No. (%)	11 (68.7)
Weight, median (Q1-Q3))	75.5 (68.4-81.2)
BMI, median (Q1-Q3))	25,3 (23.7-28.4)
Medical History:	
Hypertension, No. (%)	12 (75)
Diabetes, No. (%)	2 (12.5)
Dyslipidemia, No. (%)	12 (75)
Steatosis, No. (%)	1 (6.2)
Atrial Fibrillation, No. (%)	1 (6.2)
COPD, No. (%)	4 (28.6)
Stroke, No. (%)	1 (6.2)
EuroSCORE II^*^, median (Q1-Q3))	1,5 (0.9-3.0)
Preoperative Treatments:	
ACE inhibitor or ARB, No. (%)	12 (75)
Beta-blocker, No. (%)	9 (56.2)
Aspirin, No. (%)	11 (68.7)
Metformin, No (%)	1 (6.2)
Insulin, No (%)	1 (6.2)
Statins, No (%)	13 (81.2)
Preoperative LVEF > 50%, No. (%)	15 (93.7)
b) **Intraoperative data**	
Extracorporeal Circulation:	
Total (min), median (Q1-Q3)	70.5 (54.7-80.7)
Clamping (min), median (Q1-Q3))	59.5 (4-71)
Surgery Type:	
Valve, No. (%)	4 (25)
Coronary Bypass, No. (%)	6 (37.5)
Aortic Surgery, No. (%)	2 (12.5)
Combined Surgery, No. (%)	3 (18.7)
Myxome, No. (%)	1 (6.2)
Transfusion^**^RBCs, No. (%)	2 (12.5)
Intraoperative treatments	77 (26)
Ketoprofen, No (%)	2 (12.5)
Dexamethasone, No (%)	13 (81.2)
Norepinephrine, No (%)	16 (100)
c) **Postoperative Data**	
SAPS II^***^, median (Q1-Q3)	29 (27-37)
Peak Troponin Level (ng/l), median (Q1-Q3))	496.5 (294.5-641)
Glomerular Filtration Rate at Day 7, median (Q1-Q3))	84 (72.5-88.5)
Ventilation Duration (h), median (Q1-Q3))	6 (4-9.7)
Duration of Norepinephrine Administration (h), median (Q1-Q3))	11 (3.5-25.2)
Length of ICU Stay (day), median (Q1-Q3))	3.5 (1-5)
Total Hospitalization Duration (day), median (Q1-Q3))	9 (8-10.2)

BMI: Body Mass Index; COPD: Chronic Obstructive Pulmonary Disease; LVEF: Left Ventricular Ejection Fraction; RBC: Red Blood Cells.

* EuroSCORE II: a score used to assess the preoperative risk of cardiac surgery, subsequently yielding an estimated probability of mortality at 30 days (takes into account, among other data, age, certain medical history, critical status prior to surgery, renal function before surgery, number of previous surgeries, and number of procedures).

** Transfusion is used here as a categorical variable, including both perioperative and postoperative transfusion.

*** SAPS II: Simplified Acute Physiology Score 2; a calculated score using parameters collected during the first 24 hours of intensive care. It predicts to the probability of mortality during the stay.

### E_redox_ at CPB initiation: a brief drop

**Fig 1:**
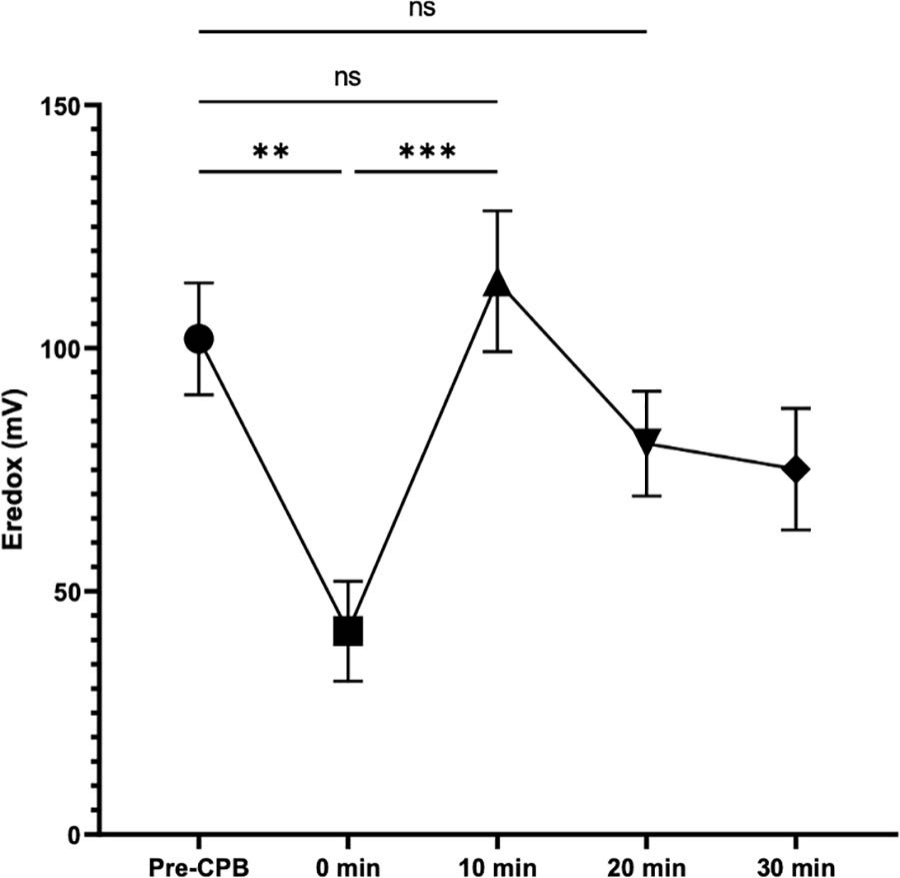
Evolution of arterial redox potential during CPB. Change in arterial redox potential (E_redox_) during CPB in millivolts (mV) relative to the baseline value before CBP initiation (Pre-CPB). Mean and standard error.

The primary objective of this research was to describe the variation in arterial whole blood E_redox_ at the initiation of CPB. Before the introduction of CPB, the E_redox_ value was homogeneous among patients with an average of 101,90 mV (+/- 11.52). We observed a significant decrease in the redox potential upon initiation of CPB (101.90 mV + /- 11.52 vs. 41.80 mV + /- 10,26; p < 0.0001) (Fig 1). Interestingly, the redox potential returns to its baseline value at the 10-minute mark and appears to stabilize without significant difference compared to the pre-CPB value (101,90 mV + /- 11.52 vs. 113.80 + /- 14.49; p = 0.98).

### Lactate and ketone bodies variations at CPB initiation

**Fig 2:**
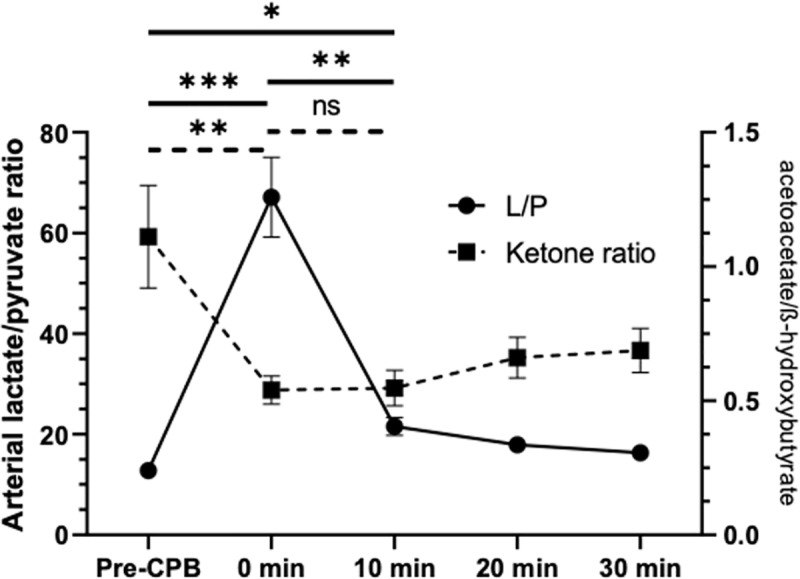
Changes in lactate/pyruvate ratio and ketone bodies ratio during CPB. L/P: Arterial lactate/pyruvate ratio. Ketone bodies ratio: acetoacetate/ß-hydroxybutyrate. CPB: cardiopulmonary bypass.

The start of CPB lead to a significant increase in lactate levels at 0 min (1.05 + /- 0.11 vs 5.38 + /- 0,38 mmol/L; p < 0.0001), and a lactate/pyruvate ratio of 12.81 (+/- 0.90) compared to 67.1 (+/- 7.94; p < 0.0001) after circulatory support (Fig 2). The blood lactate concentration returns to its baseline value within 20 and 30 minutes after CPB initiation. The lactate/pyruvate ratio remained above the baseline value at 10 minutes (21.57 + /- 1.178 vs. 12.81 + /- 0.90; p = 0,034).

The acetoacetate/ß-hydroxybutyrate ratio significantly decreased during CPB initiation (1.11 + /- 0.19 in Pre-CPB vs. 0.54 + /- 0.05 at 0 min; p = 0.0055). From 10 min to 30 min time-point, the ketone body ratio remained significative under the basal value, which is a distinctive evolution compared to other metabolic markers as lactate/pyruvate ratio and redox potential. (Fig 2).

### Concerning main hemodynamic variables

**Fig 3:**
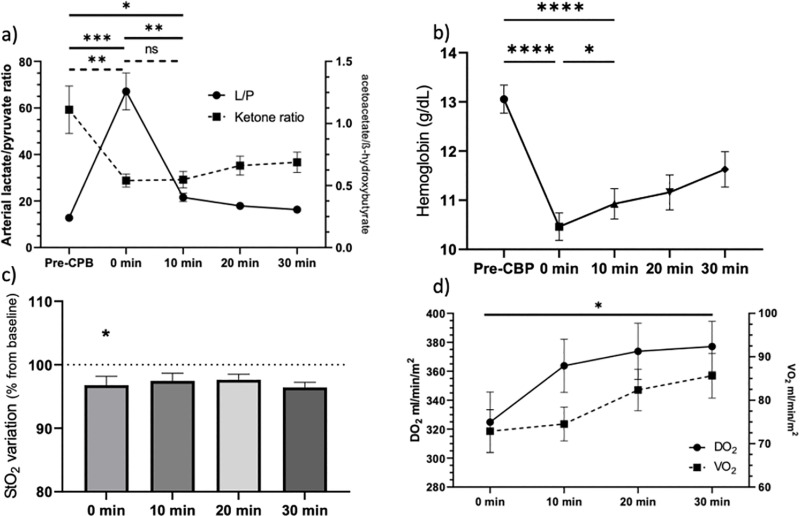
Changes in circulatory variables during CPB. **a)** Variation in systolic and mean arterial pressure and heart rate during the initiation of CPB. **b)** Hemoglobin level (g/dL). **c)** StO2 variation from baseline (Pre-CBP) measured by near-infrared spectroscopy **d)** Variations in DO2 (oxygen transportation) et VO2 (oxygen consumption) after CEC initiation; mean and standard error.

We observed a significant decrease in systolic blood pressure between the pre-CBP period and the 10-minute mark. Concomitantly, heart rate decreases with the administration of cardioplegia between 5 and 10 minutes after the initiation of CPB. The initiation of CPB did not have a significant impact on mean arterial pressure (76.4 mmHg + /- 4.8 vs 75.1 mmHg + /- 4.9; p = 0.61) as the 0 min time point was measured when the target theoretical flow rate was achieved (Fig 3a). Hemoglobin levels decreased significantly by approximately 2.6 g/dl (13.1 + /-0.3 vs 10.5 + /- 0.3) (p < 0.0001) (Fig 3b). CPB initiation also appeared to affect skeletal muscle tissue perfusion, with a significant variation in tissue saturation absolute values: 72% + /- 1.2 before CPB vs. 69.8% + /- 1.9 at 0 min (p < 0.01) (Fig 3c). DO_2_ at the 0-minute point was also significantly lower than DO_2_ at 30 minutes after the initiation of CPB (324.8 + /- 20.8 vs. 377.1 + /- 17.42; p = 0.027) (Fig 3d). All measured variables are presented in Table 2. Ten minutes after the initiation of extracorporeal circulation, the macrohemodynamic variables were stabilized without signs of circulatory failure.

**Table 2:** Descriptive table of circulatory variables during CPB.

	Pre-CBP	0 min	10 min	20 min	30 min	Pre-CBP vs 0 min p	Pre-CBP vs 10 min p
Biological variables							
E_redox_ (mV)	101.9 (11.52)	41.80 (10.26)	113.80 (14.49)	80.38 (10.77)	79.02 (22,67)	**<0.0001**	0.98
Lactate (mmol/l)	1.05 (0.11)	5.38 (0.38)	2.30 (0.15)	1.98 (0.21)	1.82 (0.17)	**<0.0001**	**<0.0001**
Pyruvate (μmol/l)	90.92 (9.75)	90.23 (7.40)	125.5 (12.54)	109.8 (9.89)	103.0 (8.13)	>0.99	**0.0217**
L/P	12.81 (0.90)	67.10 (7.94)	21.57 (1.78)	17.90 (1.41)	16.28 (1.05)	**0.0004**	**0.0045**
Acetoacetate (μmol/l)	170.2 (27.3)	200.9 (41.8)	200.7 (41.8)	221.5 (49.5	274.9 (56.6)	0.28	0.63
β-hydroxybutyrate (μmol/l)	231.2 (76.7)	498.3 (187.1)	501.9 (221.4)	514.3 (220.9)	603.8 (263.5)	0.27	0.43
Ketone ratio	1.11 (0.19)	0.53 (0.053)	0.55 (0.07)	0.75 (0.11)	0.74 (0.09)	**0.0055**	0.0079
Hb (g/dl)	13.06 (0.29)	10.46 (0.28)	10.93 (0.31)	11.16 (0.35)	11.63 (0.48)	**<0.0001**	**<0.0001**
Arterial Blood Glucose (mmol/l)	6.52 (0.38)	7.53 (0.53)	8.09 (0.43)	9.05 (0.55)	9.63 (2.22)	**0.019**	**0.0029**
pH	7.42 (0.01)	7.38 (0.01)	7.41 (0.01)	7.41 (0.02)	7.36 (0.01)	0.14	0.99
Hemodynamic variables							
StO_2_ variation (% from baseline)	100	95.21 (1.42)	98.05 (0.83)	97.27 (0.91)	96.44 (0.79)	**0.044**	0.21
SaO_2_ (%)	98.31 (0.24)	97.48 (0.49)	98.20 (0.24)	98.86 (0.21)	98.53 (0.18)	0.52	0.99
Bispectral Index	45.33 (2.13)	44.53 (2.56)	49.09 (4.02)	48.45 (3.74)	46.46 (2.60)	0.99	0.90
HR (bpm)	59.80 (4.73)	57.79 (9.47)	0	0	0	0.99	/
SAP (mmHg)	107.2 (7.27)	88.25 (6.74)	70.69 (3.42)	73.33 (2.29)	71.08 (2.71)	0.39	**0.022**
MAP (mmHg)	76.40 (4.85)	75.10 (4.94)	70.64 (3.66)	73.21 (2.25)	69.81 (2.56)	0.99	0.93
Tp (°C)	35.30 (0.18)	34.93 (0.16)	39,93 (4.29)	36.26 (0.08)	36.36 (0.13)	0.12	**0.012**
ScVO_2_ (%)	/	73.73 (1.87)	77.93 (0.86)	76.64 (0.54)	76.42 (0.55)	/	/
DO_2_ (ml/min/m^2^)	/	324,8 (20.8)	363,8 (18.4)	373,7 (19.5)	377,1 (17.4)	/	/
VO_2_ (ml/min/m^2^)	/	72.85 (4.97)	74.51 (3.88)	82.37 (4.78)	85.62 (5.16)	/	/
EO_2_	/	23.52 (1.73)	20.69 (0.90)	22.00 (0.49)	22.64 (0.64)	/	/

Quantitative variables: mean (standard error); E_redox_: arterial redox potential; L/P: arterial lactate/pyruvate ratio; StO_2_ variation: tissue oxygen saturation variation from baseline (Pre-CPB); SaO_2_: arterial oxygen saturation; Hb; hemoglobin; SAP: systolic arterial pressure; MAP: mean arterial pressure; Tp: central body temperature; Ketone bodies: acetoacetate/β-hydroxybutyrate ratio; ScVO_2_: central venous oxygen saturation; DO_2_: oxygen delivery; VO_2_: oxygen consumption; EO_2_ = VO2/DO_2_ ratio. Ketone ratio: Acetoacetate/β-hydroxybutyrate P value of ANOVA and Mann-Whitney.

## Discussion

In this study, we demonstrated that CBP initiation is associated with a significant decrease in arterial E_redox_, and that the return to baseline was faster when monitored using the E_redox_. We also observed an increase in the lactate/pyruvate ratio and a decrease in the acetoacetate/β-hydroxybutyrate ratio. The initiation of CPB led to significant macro- and micro-hemodynamic disturbances.

To characterize the onset of hemodynamic instability upon the initiation of CPB, we observed disturbances in macrohemodynamic variables. It included a decrease in systolic arterial pressure (concomitant with cardioplegia), a reduction in hemoglobin levels, DO_2_ values at 0 minutes versus 30 minutes, and a significant decrease in tissue oxygenation. These disturbances fall within the usual ranges for the initiation of CPB [10]. In our view, they indicate that the implementation of CPB is associated with hemodynamic instability that may lead to circulatory failure in the patient’s tissues [5,11–14]. We aimed to confirm this circulatory failure by precise measurement of metabolites that are not routinely accessible but are early indicators of shock states. We regularly measured the arterial concentration of lactate, pyruvate, acetoacetate, and β-hydroxybutyrate. These measurements allowed us to obtain a more precise description of cytoplasmic and mitochondrial redox state. We observed a rapid disturbance of both systems upon the initiation of CPB. Firstly, we noted a significant increase in lactatemia while pyruvatemia remained stable. These results are already described in the literature [15]. A lactate/pyruvate ratio greater than 24 is associated in the literature with increased mortality in septic shock. It is a marker of hypoperfusion [16]. During shock, the lactate/pyruvate ratio increases in the blood as well as in adipose tissue and skeletal muscle [17,18]. This indicates a metabolic shift towards anaerobic glycolysis and lactate production [19]. The impaired elimination of lactate can be also explained by a disturbance in hepatic metabolism. This explains the slower return of lactatemia to its baseline value, in contrast to the E_redox_, which appears to be more reactive. This hypothesis seems to be supported by the decrease in the ketone ratio [20]. The literature agrees on the microcirculatory disturbances during CPB. These are reflected by the increased production of lactate, alterations in tissue saturations, impairment of arteriolar vasoreactivity, and a decrease in the number of perfused capillaries [5,13,14,21].

The variations in the acetoacetate/β-hydroxybutyrate ratio are characteristic of a metabolic shift towards ketolysis and the production of acetyl-CoA [22]. This is a marker described in the literature as indicative of circulatory failure. Ketones are an alternative energy source for tissues, particularly the brain. The rapid variations in the acetoacetate/β-hydroxybutyrate ratio are characteristic of the onset of stress in the body [23,24]. It is also an indicator of the hepatic response to the insult. The abrupt decrease in the ratio after the initiation of CPB indicates the release of β-hydroxybutyrate by hepatic mitochondria. Once in the systemic circulation, β-hydroxybutyrate becomes a source of ATP for the tissues [22]. This decrease has been observed in several studies [25,26].

Intraoperative lactatemia is a good predictor of patient outcomes [27]. The higher it is, the greater the risk of mortality. In contrast to lactate, although ketone bodies play a role in highlighting hepatic dysfunction or hypoxic processes, they are not correlated with patient outcomes [25]. The ketone body ratio values are comparable to those found during septic shock and cardiogenic shock [28]. The decrease in the ketone body ratio below 0.4 during CPB is correlated with the elevation of transaminases postoperatively [25]. However, our study did not reach such low levels, and the patients did not exhibit elevated hepatic enzymes postoperatively.

Ultimately, we demonstrate through precise biological variables that are not accessible in routine practice that there is a phase of hemodynamic instability associated with tissue circulatory failure during the initiation of CPB. The ability to detect circulatory failure is of paramount importance for the proper monitoring of patients in the cardiac surgery operating room [29]. Therefore, we developed a system to measure the E_redox_. The metabolic disturbances in tissues during circulatory failure are numerous and lead to the release of various metabolites. The measurement of lactatemia, which is available in routine practice, presents significant limitations [30].

The measurement of arterial whole blood E_redox_ during CPB is an innovative approach that has not been previously described in humans. We observed a marked reduction in E_redox_ during CBP initiation, with consistent patterns among patients as indicated by the observed standard deviations. The measurement of global redox state in whole blood provides a more comprehensive approach than measuring circulating metabolites, which are limited in their knowledge and subject to technological constraints. This global measurement is thus likely to be more sensitive to tissue hypoxia. Few studies have investigated E_redox_ in humans, and previous measurements have been limited to plasma samples [4,15]. Measuring only plasma E_redox_ cannot represent the systemic redox state of a patient as it disregards the redox contribution of blood cells. Red blood cells play a crucial role in the redox balance of blood. Therefore, arterial whole blood E_redox_ appears to be a promising technique for detecting and monitoring mitochondrial dysfunction induced by circulatory failure. We demonstrated that the arterial E_redox_ decreases significantly at the initiation of CPB and appears to coincide with the onset of circulatory failure. This E_redox_ rapidly returned to baseline after achieving satisfactory macrohemodynamics status. Several spectroscopic techniques, such as NIRS and SvO₂, highlight increased tissue oxygen extraction in situations of circulatory failure. However, these methods primarily reflect tissue adaptation to adverse conditions rather than direct tissue injury.

This study opens numerous perspectives for both research and potential clinical applications. The practice of anesthesia and intensive care is guided by advances in therapeutic strategies to combat tissue hypoxia, regardless of its origin. Therefore, tools for exploring the correction of redox imbalance are appealing for diagnosing and stratifying patient risk. In terms of research, adaptation of the measurement system could enable continuous monitoring of this parameter in cardiac surgery and intensive care patients, allowing continuous assessment of blood redox balance and early detection of circulatory failure. The technique appears to be complementary to cardiac output monitoring. Evaluating mitochondrial function would be a valuable way to assess the match between oxygen demand and transport. The E_redox_ could enable us to identify different variability profiles not observed in this study, particularly in more severe patients. The study of E_redox_ is meaningful only if distinct profiles exist, allowing for the proposal of therapeutic adaptations to restore tissue oxygenation. Unlike lactates, where clearance is sometimes impaired, the rapid correction of E_redox_ could provide greater responsiveness to clinicians. These hypotheses will need to be confirmed in larger-scale studies.

E_redox_ provide informations on the sum of electronic exchanges among all the redox couples at thermodynamic equilibrium contained in the sample. Then the whole blood redox status must be evaluated with a delocalized device easely usable in the operating room, which implies a reagentless method, without sample preparation and providing realtime results. The global tests as developed today do not meet all these requirements. Moreover, they do not consider the latency phase of certain reactions, kinetically disadvantaged with the metallic complexe, in particularly the liposolubles antioxidants such as alphatocopherol, and the intervention of redox molecules which are not antioxidant, like proteins or lipids.

The main limitation of this study is the small sample size, with only 21 patients included, of which 16 had measurements of E_redox_. However, this study was exploratory, aiming to validate the feasibility of the technique and its correlation with lactate levels.

The patients included in the protocol had an average Euroscore II of 1.5%, which is relatively low compared to larger cohorts (3.95%) [31]. The SAPS II score was on average low at 29, compared to an average of 39 in cardiac surgery literature [32]. One patient in the cohort had type II diabetes and was on insulin and metformin. Thirteen patients were treated with statins prior to inclusion. Thus, the patients in the study presented a low risk of postoperative complications and, consequently, a low risk of mortality in the context of cardiac surgery. The patients in this study thus appeared to be less comorbid than the usual population undergoing cardiac surgery. The effects observed upon the initiation of CPB do not seem to be related to a particularly altered state of the study patients. In this preliminary study, we deliberately focused on elective surgeries. These procedures enable the measurement of redox potential without prior physiological stress. The baseline redox potential value remains undefined. In contrast, emergency surgeries carry the risk of pre-existing redox potential disturbances before the intervention.

The presence of treatments (insulin, metformin, statins) and conditions (diabetes) that could potentially alter metabolism did not affect the arterial blood E_redox_. We hypothesize that this is due to the intensity of the insult related to the implementation of CPB. CPB profoundly disrupts tissue metabolism, as evidenced by the disturbances in lactate/pyruvate and acetoacetate/β-hydroxybutyrate ratios. It is established that statins may help mitigate the effects of hypoxia on tissues [33]. As expected in this population, only one patient was not on statin therapy before surgery. Their redox profile was comparable to that of the other patients. This could also be explained by the severity of the insult associated with the initiation of CPB.

The purpose of CPB is to replace the patient’s blood flow, resulting in a very brief period of circulatory failure. The ideal characterization of this mismatch between oxygen supply and demand would involve continuous measurement of mixed venous oxygen saturation (SvO_2_) and cardiac output prior to CPB initiation, allowing calculation of tissue oxygen consumption. However, these measurements were not performed in this study. Nonetheless, we observed concordance among variables, suggesting that CPB initiation is indeed associated with circulatory failure (lactate/pyruvate ratio, systolic blood pressure, near-infrared spectroscopy, hemodilution, ketone bodies). The existing literature also leaves little doubt on this matter. In the future, it would be relevant to conduct similar research in other models of circulatory failure.

## Conclusion

Arterial whole blood E_redox_ is a novel variable that holds promise in the detection and monitoring of blood metabolic perturbations during CPB initiation. Its assessment during CPB initiation could provide valuable insights into the patient’s circulatory status, as the E_redox_ appears to be more sensitive than lactate for monitoring circulatory insufficiency. Further larger-scale studies will be necessary to confirm the relevance of this new variable in perioperative and intensive care monitoring.
